# Explaining “What for” in Motion Analysis Research: A Proposal for a Counterfactual Framework That Is Slightly Different From the Theory of Causation

**DOI:** 10.3389/fspor.2021.699322

**Published:** 2021-11-10

**Authors:** Leon Omura, Senshi Fukashiro, Shinsuke Yoshioka

**Affiliations:** ^1^Department of Life Sciences, The University of Tokyo, Tokyo, Japan; ^2^Research Fellow of the Japan Society for the Promotion of Science, Tokyo, Japan; ^3^Japan Women's College of Physical Education, Tokyo, Japan

**Keywords:** biomechanics, motion analysis, fitness for purpose, teleological thinking, movement technique

## Abstract

In motion analysis research, the methodology for estimating the physical processes of human movement is highly developed, but the methodology for interpreting such data is relatively undeveloped. One of the aims of this paper is to demonstrate the importance of developing a conceptual basis for interpreting data about the physical processes of body movement. In this conceptual study, one topic was discussed as a central question: what it means to answer the question what a certain movement technique is aimed for. We first introduced the distinction between explanations from the perspective of causes and explanations from the perspective of purposes as a mode of explaining events, and pointed out the importance of explanations from the perspective of purposes. We next argued that by taking the perspective of whether a given movement technique leads to a desired outcome in comparison to other movement techniques, we can expect to interpret what a given movement technique is for based on objectively observable information rather than the subjective intentions of the athlete. In addition, we discussed how the criterion movement patterns should be defined when assessing the fitness for purpose of a given movement technique in terms of its consequences. In this regard, our argument is that it is necessary to take into account that the exact same movement pattern cannot be performed every time, even for the same motor task, and that there are multiple options for how to define the set of possible movement patterns that can be performed. Our discussion reveals the peculiarity of grasping the meaning of movement techniques, and therefore suggests that there is a substantial need for motion analysis researchers to deepen their conceptual analysis to understand the nature of this issue.

## Introduction

In motion analysis research, a subfield of biomechanics, research methods for estimating the physical states of the musculoskeletal system during human movements are highly developed (Winter, 2009; Robertson et al., 2014). In contrast, there seems to be much room for development regarding the methodology of interpreting the estimated data of physical states. However, this issue is more elusive than the description of human movements in terms of physical processes. As an initial step toward development, it would be useful to conduct a conceptual analysis to discern what needs to be done to move the issue forward. One of the aims of this paper is to illustrate the necessity and utility of such a conceptual analysis in the field of motion analysis research.

To achieve this goal, we will thoroughly explore what needs to be investigated to answer the question of what benefit a particular movement technique offers; that is, what is the fitness for purpose of that movement technique. We focus on this topic because, although this topic would be deeply related to one of the central interests of sports biomechanics, what are the key technical factors of superior sports performance (Bartlett, 1997; Chow and Knudson, 2011), the conceptual basis for grasping it appears to be inadequately established.

The discussion in this study comprises two major parts. The first is to define the difference between explaining why a particular movement technique is employed and explaining the causal mechanism from which a particular movement pattern is generated. We believe that we need to establish a conceptual framework that falls somewhere in between these two explanatory modes; that is, while explaining the fitness for purpose of movement techniques is similar to each explanation mode in some respects, it also differs in some important points. Therefore, we need to start our discussion by clarifying the relationship between the two explanation modes and explaining the fitness for purpose of movement techniques.

The second is a consideration of more specific issues that may arise when attempting to grasp the fitness for purpose of a certain movement technique in actual motion analysis research. We specifically concentrated on presenting a conceptual framework for comparative inference that is similar in some respects to, but different in important ways from the framework used to specify causal relationships between events. We believe that these considerations provide indications of the type of data analysis needed in the future.

## Preliminaries

Before proceeding to the detailed discussion, we shall briefly clarify some of the terms that will be used repeatedly in the following discussions. First, the term “movement technique”; we use this term in a broader sense. The term “technique” would typically refer to a set of characteristic patterns in which multiple elements cooperate. We use the term to refer not only to such, but also to the behavior of more localized body elements; for example, a knee extension torque is set to 150 Nm in a particular movement phase. This is because in motion analysis research, the local behavior of a specific element is often focused on as a characteristic feature of the movement. Next, the term “movement pattern” is used to refer to all mechanical states that constitute a certain movement sequence. Finally, we use the term “movement outcome” to refer to the evaluation given to a movement sequence, such as the success or failure of a movement task, a win or lose, or a record.

## Questions About “How It Occurs” and “What It Aims for”

From natural phenomena to everyday events, we often ask ourselves why they occur (or occurred). In this section, we introduce a basic contrast between the two modes of explanation in the human and biological sciences as an answer to the question “why does it arise?.” This contrast will aid in the following discussion.

We can answer the question of why an event occurs in the form of “how it occurs.” In motion analysis research, the question of why the observed motion of a body part occurs is often answered from the perspective of the combination of muscle forces or torques (and other forces such as gravity) causing the motion (Nott et al., 2010). We can further investigate how such muscle forces are generated. In this case, the explanation can be provided in terms of the physiological processes inside the body that lead to the generation of muscle forces. Another explanation is in terms of “what it aims for.” For example, the explanation that a certain muscle force acts to stabilize a joint (Flaxman et al., 2012).

This contrast between the two modes of explanation follows the traditional distinction between objective “causes” and subjective “reasons” in the disciplines that focus on human action (Anscombe, 1957). The former aims to explain the behavior of objects in physics and other natural sciences. There is a belief that the discipline concerning human actions should eventually be replaced by an objective explanation from an external point of view similar to that of physics (Churchland, 1981). The latter, however, has been emphasized by those who believe that the application of natural scientific methods alone is insufficient to understand human action, and that it is necessary to inquire into the meaning of action by the actors themselves. That is, the reason a person acted in a certain manner becomes understandable to others only when the subjective intention and purpose of the action are explained (von Wright, 1971). For example, when we ask why a person is standing with his/her hand up, we can say that it is because there is a physical state of the body that allows him/her to stand with his/her hand up, but the answer that he/she is doing so to pick up a cab is more likely to give us a sense of acceptance of his/her behavior.

A similar contrast has traditionally existed in the biological sciences. The behavior of organisms may appear to be purpose-oriented, but there is also a view that these behaviors can eventually be replaced by descriptions from the perspective of physical processes, and therefore we should aim in this direction (Mahner and Bunge, 1997). However, it is also believed that explanations from the perspective of purpose, known as teleological explanations, from the Greek “telos,” meaning purpose or end, will continue to be necessary in the field of biological sciences (Canfield, 1964).

## Differences in the Ability to Determine What Occurs

In this section, we highlight an important difference between the two modes of explanation. We discuss this because the two modes of explanation are similar in some respects, and their differences are not always apparent.

It should be noted that some believed that the two modes of explanation are both explanations of causes. Aristotle believed that there were four types of answers to the question of why things are the way they are; he considered material cause, formal cause, efficient cause, and final cause to be the causes of things. As an example, consider how to explain the existence of the biarticular muscle. We can explain the biarticular muscle from the perspective of what it is materially composed of (material cause). We can also say that the morphological feature of straddling two joints is an essential feature shared by all biarticular muscles (formal cause). The reason why the biarticular muscle exists in its present form can be explained by the history of biological evolution (efficient cause). The reason for the existence of the biarticular muscle could also be explained in terms of what it is for, for example, that it exists to make energy transfer to the terminal limb more efficient (Elftman, 1940) (final cause). However, Aristotle's definition of these four causes is quite strange for the modern age. Therefore, it should be noted that the examples presented above do not reflect the original meanings of the four causes but rather a reconstruction of the framework from the perspective of a modern scientist. Among them, the efficient cause explains from the perspective of what is the first beginning of the existence or change of motion of a thing, while the final cause explains from the perspective of what is the final destination of a thing (Ross, 1936). The former is similar to the explanation of motion by forces in physics, whereas the latter is similar to the teleological explanation. Since the success of Newtonian mechanics, only explanations from the former point of view have commonly acquired the status of genuine explanations of causes, and the notion that purposes are not true causes has become widespread (Rosemberg, 2000). In the following section, we also use the word cause to include only the former. However, even today, there are those who regard explanation from the perspective of purpose as a type of explanation by causes (Rabins, 2013). We might also use explanation by cause and explanation by purpose in our daily conversation without making a strict distinction.

In the remainder of this section, we discuss why the notion that only an efficient cause is a genuine cause has a strong influence, and why it is sometimes intuitively felt to be shown a cause, even from an explanation from the perspective of purpose. We believe that this makes it difficult for motion analysis researchers to recognize the difference between the two modes of explanation.

It is sometimes pointed out that a cause has the nuance of something that enforces its consequences (Kutach, 2014). In addition, it is suggested that explaining the occurrence of an event consists of providing information that changes more “surprising facts” into “a matter of course” (Peirce, 1935, 1974). In this regard, there is a substantial difference between an explanation of how an event occurs and an explanation of what the event aims for. If we can fully specify the elements that cause an event, it will be clear what type of event will occur. For example, considering a forward dynamics simulation using a musculoskeletal model, once the combination of forces acting on the skeletal system (i.e., the causes) is identified, the resulting acceleration (i.e., what occurs due to the causes) can be uniquely predicted. That is, the complete identification of a causal process allows us to be certain that there is no possibility of an event other than the event realized by that causal process occurring. Hence, showing the causes seems to have strong explanatory power for the occurrence of events. In contrast, determining the accelerations desired to be generated in the skeletal system does not necessarily uniquely identify the combination of muscle forces that will generate them. In this sense, merely grasping what it aims for does not give us a firm conviction of the occurrence of that specific event.

However, in the analysis of human movements, there are likely to be cases where it is possible to identify to some extent what type of behavior occurs by considering it from the perspective of what the behavior aims for. For example, when performing a reaching task, a particular hand trajectory is highly preferred, even though there are numerous possible hand trajectories that can reach the target position (Morasso, 1981). Such characteristic hand trajectories can be reproduced theoretically by assuming that the actor behaves in a manner that optimizes certain types of objective functions (Viviani and Flash, 1995; Harris and Wolpert, 1998; Nakano et al., 1999). In such cases, it may seem that the behavior is uniquely determined by what one is aiming to achieve. Here, we find a similarity to the property of causal explanation, as pointed out above. However, it is not always possible to determine exactly what events will occur by grasping what the behavior is aiming for. This is because some motor tasks may be accomplished in more than one way, with no consistent preference among individuals (Bartlett et al., 2007). Therefore, explaining the behavior of a body part in terms of the goal of a motor task should not be regarded as entirely equivalent to a causal explanation.

## Reasons Why We Need to Explain “What It Aims for” in Motion Analysis Research

We intend to defend the notion that a certain type of teleological thinking is sometimes necessary in motion analysis research. However, the necessity of teleological thinking is not self-evident. For example, considering the discussion in the previous section, once we know the exact causal process that generates a movement, it becomes clear what specific material and physical conditions need to be fulfilled to replicate that movement. However, merely knowing what goals to achieve does not allow us to specify what physical processes need to be prepared to realize the desired movement. Hence, from a practical standpoint, it appears more useful to know the details of how (from what causal processes) the movement occurs. Therefore, in this section, we consider the rationale behind this.

First, the reason often mentioned as a benefit of teleological thinking cannot successfully justify its utility in motion analysis research. One of the practical benefits of using teleological thinking is that it helps to reduce our thinking costs (Dennett, 1987). Even if we do not know the exact causal mechanism by which an event occurs, we can sometimes accurately predict what events will occur by utilizing a teleological perspective. For example, it would be very difficult to explain the seemingly irregular flight trajectory of a hawk in terms of the complex material and physical processes of living organisms, but it would be much easier to explain the flight trajectory of a hawk in terms of the purpose of flying to track its prey. Similar savings in thinking costs can be expected when we are able to intuitively accept the actions of others. When the purpose of someone else's action is explained, we feel that we are able to understand that action without needing to know about the physiological processes involved in its execution. This kind of quick understanding is possible when one knows immediately that he/she would act in such a manner if he/she had a similar purpose. That is, the utility of understanding a situation from the perspective of its purpose increases when the means to achieve a certain goal can be found without deliberation.

However, in motion analysis research, it is difficult to intuitively grasp the mechanical effect of a certain muscle force owing to the complexity of the conversion relationship from force to whole-body acceleration (Thelen et al., 2013). Thus, even if we can grasp the purpose of a movement, it does not necessarily imply that we can easily grasp the means to achieve it. Therefore, it seems difficult to justify teleological thinking in terms of reducing thinking costs.

In motion analysis research, it seems that teleological thinking should be implemented not because it helps to reduce our epistemological burden, but because it is necessary for a deeper understanding of movement techniques of interest. Many motion analysis researchers are interested in whether the employment of a certain movement technique is beneficial in achieving high performance in a particular sport. To assess whether a certain movement technique is beneficial, it seems insufficient to identify the causal processes of body movements when it is executed. Instead, it seems necessary to consider its suitability for the purpose or functional requirements of motor task. This is where the need to understand movement in relation to its purpose emerges.

Furthermore, we need to consider why we should understand whether and why certain movement techniques are beneficial. Certainly, considering these questions may satisfy our pure intellectual curiosity, but its practical significance is not immediately clear. We believe that understanding movement techniques from a teleological perspective has important practical implications. It is grounded by the fact that the strategy of merely imitating specific movement patterns exhibited by an elite athlete seems to be less effective. Even among top-level athletes, there is diversity in the movement techniques they employ (Whiting et al., 1991; Schöllhorn and Bauer, 1998). Hence, the suitable movement pattern relative to one's own physical characteristics may vary among players (Glazier and Mehdizadeh, 2019). In addition, in motor tasks such as interpersonal competitions, which are characterized by intense fluctuations in external situations, it is desirable that one does not always execute the same movement pattern, but rather executes an appropriate movement pattern in response to changes in the external situation.

In light of these difficulties, the strategy of attempting to copy the same movement patterns performed by athletes who exhibit highly competitive performances may not always be effective. In such cases, it is important not to attempt to replicate a specific movement pattern exactly as it is, but to understand what principles enable the efficient execution of the motor task, and then to utilize those principles in a manner that is appropriate for each physical characteristic and situation. If that is the case, it is assumed that it will be very important to understand in what sense a certain movement technique is fit for purpose.

## Requirements Specific to Motion analysis Research When Considering Fitness for Purpose

The question of how to explain “what a thing is for” has been discussed in various academic areas in which it is necessary to view things in relation to their purpose and function, such as human actions, organs of living things, and product design (Dretske, 1988; Far and Elamy, 2005). The development of such discussions must have differed depending on the background of each research area. Likewise, in motion analysis research, to answer the question of why a certain movement technique is employed, it is necessary to adjust the framework to match our intellectual interests and the available research tools. In this section, we propose the following three points to be considered when attempting to explain “what a certain movement technique is for” in motion analysis research. A preliminary consideration is then provided in the discussion regarding what types of ideas are likely to be needed to satisfy them.

First, we consider that it is necessary to establish a framework that allows us to infer “what a certain movement technique is for” using information only at a standard level in biomechanical motion analysis research, such as joint motion and its associated forces and torques. The reasons for human social action are often explained in terms of what consequences were intended (Bratman, 1987). This seems to be a useful way of thinking when the action we are interested in is such that the actors themselves have an awareness of what they are doing. In addition, in the design of artifacts, we can expect to define what a certain component is for based on the intention of the person who designed it (Vermaas and Houkes, 2006). However, in the case of human movement, the behavior of all the elements that constitute a movement does not necessarily reflect the intentions of the athlete him/herself or a person who plays the role of a designer, such as a trainer. Therefore, it is not always easy to map information at the biomechanical level to information at the human intention level. In such cases, defining what a certain movement technique is for in a hierarchically autonomous manner in biomechanical movement analysis would be beneficial in expanding research options.

Second, it is necessary to develop a framework that can sensitively assess the fitness for purpose of a movement technique. The movement techniques used by experts are expected to be beneficial in achieving good movement outcomes in motor tasks. However, motion analysis researchers may also be expected to critically improve these techniques occasionally, rather than fully trusting the efficacy of existing techniques used by experts. Therefore, when considering what a certain movement technique is for, or what fitness for purpose it has, it is desirable to sometimes conclude that it is not so beneficial.

Third, it is desirable to be able to capture what a given movement technique is for, even when it cannot be explained by simply reducing it to some mechanical quantities. It is not always necessary to employ a unique way of thinking to understand a certain movement technique in relation to its purpose or functional requirements. For example, “During the stance phase of walking, certain muscles of the supporting leg act vigorously for supporting the posture of the body against gravity” (Anderson and Pandy, 2003) seems to satisfy the question of what a certain muscle force is acting for. This can be explained by adding a relatively simple interpretation to the analysis of the causal relationship between force and acceleration obtained using the equations of motion, that is, the acceleration effect produced by a force is considered to be the purpose or function for which the force is acting. However, this approach is not applicable to all problems. Issues arise when the analysis of instantaneous mechanical relationships does not immediately reveal the global benefits of a given movement technique. Such a situation may be more familiar in cases in which the relationship between the goals and means of movement is more complex, such as in interpersonal sports. Therefore, in terms of general applicability, we need a framework that can capture the fitness for purpose of employing a certain movement technique from a different perspective than the mechanical effects at a given instant.

We argue that the above three requirements can be satisfied by considering that the explanation of “what the movement technique is for” is an explanation in terms of “whether it increases the probability of a more desirable movement outcome than if alternative candidate movement techniques is employed.” This notion is influenced by an idea to concisely define the fitness of an organism's traits for their environment: “x is fitter than y if and only if x's traits enable it to solve the ‘design problems’ set by the environment more fully than y's traits do” (Dennett, 1995).

One characteristic of this notion is that it interprets the question “what for” as something that should be answered in terms of “what will be obtained as a result” rather than “what the intention was.” In this manner, we can expect to provide explanations based on information at the biomechanical level. Another feature is to capture the significance of a movement technique in relation to the execution of a motor task through comparison with cases in which other movement techniques are employed. By including such a perspective, it is possible to consider whether a particular movement technique is more suitable than other good movement techniques. Thus, based on the definition we have provided, the meaning of a given movement technique in relation to the execution of a motor task fluctuates depending on whether a better movement technique exists. This is compatible with the notion that, in the context of the pursuit of higher sports performance, it seems important to have a perspective on what the employment of a particular movement technique can achieve in comparison to other good movement techniques. Furthermore, the approach of considering the differences in consequences relative to situations in which other movement techniques are employed is applicable even in cases in which the analysis of instantaneous mechanical relationships is not useful. For example, if we can examine the difference in the end phase of a movement when a certain movement technique is employed at the beginning of the movement phase compared to other movement techniques, it becomes possible to relate such temporally distant events. Conveniently, at this time, we are relieved of the burden of connecting two events with a mechanical equation.

## Is the Framework of Intervention Experiments for Identifying Causal Relationships Directly Applicable?

In this section and subsequent sections, we will discuss how to determine whether a given movement technique is fit for purpose in order to better perform a motor task. In the previous section, we explored what kind of framework is needed to think about the purpose of movement techniques without having to infer the subjective intentions of actors. Therefore, answering the question, “what are the consequential benefits of employing a certain movement technique compared to competing movement techniques?” is somewhat similar to answering the question, “what are the prospective causal effects of a new prescription of that movement technique?.” However, we believe there is an important difference between thinking according to the definition given in the previous section and identifying causal relationships; thus, a careful distinction needs to be made. In this section, referring to the framework of counterfactual thinking and intervention experiments that have been used to estimate the existence of causal relationships, we discuss why they should not be directly applied to the assessment of fitness for purpose of movement techniques.

The intervention experiment is a very important tool for identifying causal relationships. It is well-known that the existence of a correlation between variables does not necessarily imply a causal relationship. This is because there can be events that are causally unrelated but only temporally coincident (Pearl, 2000). Further, whether the correlations that exist are derived from genuine causation or spurious correlation cannot be discerned by passive observation alone. In such a case, if a prior event has a causal relationship with another subsequent event, manipulation of the former would change whether and how the subsequent event occurred. Such counterfactual thinking of what would occur if one event were replaced by another has been incorporated into actual scientific research using the method of the intervention experiment (Woodward, 2003). In an intervention experiment, it is important to make the background conditions between the control and intervention conditions as homogeneous as possible, with the exception of the intervention itself. By doing so, it is possible to specify that the intervened event is causally related to the change in the event that occurred after the intervened event (Yeadon, 2005).

We need to be cautious about directly applying such a framework of intervention experiments to estimate causal relationships in order to assess the fitness for purpose of movement techniques. One reason is that it is almost impossible to conduct interventions that address only a specific element of human movement (Bobbert and van Soest, 1994). In addition, there is a more essential issue. When considering the fitness for purpose of a movement technique, it is necessary to consider that the employment of a certain novel movement technique requires an overall reorganization of the movement pattern. If only the characteristic aspects of a given movement technique are incorporated, and no overall readjustment of the movement pattern is made, the movement outcome may decrease rather than improve (Bobbert and van Soest, 1994; Glazier and Mehdizadeh, 2019). It is expected that this readjustment will require a long period of practice (De Rugy et al., 2012; Hagen and Valero-Cuevas, 2017). That is, even if the movement technique of interest is effective in improving performance after overall readjustment of the movement pattern, such a conclusion could not be reached if a pure intervention experiment is directly applied.

## Selection of Movement Techniques to be Compared

In section Requirements Specific to Motion Analysis Research When Considering Fitness for Purpose, we proposed that in assessing the fitness for purpose of a movement technique, it would be useful to adopt the criterion of whether employing the movement technique increases the probability of obtaining a better outcome. Notably, this criterion also contains a type of counterfactual thinking that would occur in a different situation. This suggests that although the framework of intervention experiments for identifying causal relationships should not be simply applied, a different counterfactual thinking framework needs to be established.

To this end, there are at least two points about the criteria presented in section Requirements Specific to Motion Analysis Research When Considering Fitness for Purpose that should be more refined. One is what alternative movement techniques indicate. The other is what the consequences of employing a certain movement technique indicate. These meanings should be carefully considered, as they affect the assessment of the fitness for purpose of a movement technique. In this paper, we predominantly discuss the second point. However, the second point is difficult to understand without reference to the first point. Therefore, in this section, although we will not discuss the first point in detail, we organize an outline of the issues to be considered in the future and provide provisional comments.

The most important point that needs to be considered regarding the first issue is whether the “comparison with alternative movement techniques” should be made with a single or multiple alternative movement techniques. This is an issue derived from the fact that there may be a large number of candidate alternative movement techniques for comparison. Therefore, there is a matter of selection as to which movement techniques should be compared.

One possible approach is to define “if alternative movement techniques are employed” as the assignment of several candidate alternative movement techniques according to their probability of being employed. Under this policy, it is further necessary to determine the reference population in specifying “candidate alternative movement techniques and the probabilities that they are employed.” This is because, for example, it is assumed that there is a difference between the existing movement techniques and their probabilities of employment in a group that includes novices to top athletes and those in a group of only top athletes. A potential advantage of this approach is that it allows comparison with the remainder of the whole to be considered collectively, which may be beneficial in terms of summarizing a large amount of information. In addition, this approach may be particularly useful if the only interest is the relationship between the employment of movement techniques and the eventual movement outcome. However, if we wish to investigate even the intermediate process of movement, confusion may arise due to the mixing of cases in which different movement techniques are employed.

The notion that only a particular movement technique should be selected for comparison is also worth considering. With this approach, it is not necessary to perform the likely time-consuming task of examining the extent to which the movement technique has been employed in the reference population. Furthermore, there will be relatively less confusion if we wish to analyze the intermediate processes of movement and investigate why a movement technique is (or is not) fit for purpose. It should be noted, however, that when a single movement technique is selected for comparison, the assessment of the fitness for purpose of the technique of interest will be affected depending on which technique is included as an alternative candidate.

Supposedly, there is no single standard that should always be applied to the issue discussed in this section. Nevertheless, it is clearly an issue that needs to be considered carefully, as the assessment of the fitness for purpose of movement techniques of interest will vary depending on how one regards this point. In the future, we will need to formulate the strengths and limitations of each of the possible approaches and their specific methods of application, and to be self-aware and explicit about which is being adopted. This will avoid unnecessary confusion among studies based on different criteria.

## Possibility of Assessing Fitness for Purpose of Movement Techniques According to the Movement Pattern That Best Completes a Motor Task

As pointed out in section Is the Framework of Intervention Experiments for Identifying Causal Relationships Directly Applicable?, when considering the types of movement outcomes that will occur when a certain movement technique is employed, it is necessary to consider the movement patterns adjusted for each movement technique to allow for fair comparison. Therefore, in sections Possibility of Assessing Fitness for Purpose of Movement Techniques According to the Movement Pattern That Best Completes a Motor Task and Movement Patterns That Can Be Performed, we will attempt to define which movement patterns should be considered as those that are performed when a certain movement technique is employed. To avoid excessive complexity in the following discussions, the point discussed in the previous section is assumed using only one alternative movement technique as a comparator.

First, the movement pattern that achieves the best movement outcome when employing a certain movement technique should be regarded as the movement pattern that is performed under that condition. Such a notion is likely already implicit in research using simulation-based optimization. For example, one study investigated whether the presence of bi-articular muscles is suitable for the purpose of increasing vertical jump height (van Soest et al., 1993). In this study, a normal musculoskeletal model and a model in which the bi-articular gastrocnemius muscle was replaced by a monoarticular muscle of equivalent volume were examined by comparing the movement patterns that achieved the highest jump heights.

If this notion can be justified, we need consider only one movement pattern that will occur if a certain movement technique is employed, and this simplicity has a certain appeal. However, this approach had some limitations. For example, the practical difficulty of determining the movement pattern that achieves the best movement outcome. To identify the movement pattern that achieves the highest performance when employing a certain movement technique, it is desirable to conduct a simulation study, but it is necessary to consider the technical limitations of the current optimization simulations. The identification of a neural input in the musculoskeletal model that optimizes the objective function (movement outcome) would constitute a big step toward making this approach feasible. However, when the degrees of freedom of the model are too large or the target movement is complex, the current standard is to search for neural input patterns that track the measured kinematic patterns, mainly because of the large computational burden (Ehsani et al., 2016; Lin and Pandy, 2017). Therefore, there is a limit to the practical applicability of using optimization simulations to assess whether one movement technique leads to a more desirable outcome than another.

Furthermore, some more principled problems can be identified. For instance, the approach does not consider the fact that even experts cannot repeat the exact same movement pattern every time (Davids et al., 2003; Preatoni et al., 2013). This is problematic, assuming that there are movement techniques with the advantage of resistance to error (Hiley and Yeadon, 2003). Furthermore, it is considered inadequate as a framework for capturing situations in which the outcome of the movement is affected by changes in the external environment, such as wind direction, ground conditions, and the behavior of allied or opposing players in interpersonal or collective sports. For example, in interpersonal sports, it would not be useful to assume a single movement pattern when employing a certain movement technique, because the type of movement pattern that is fit for purpose should vary depending on how the opponent moves.

## Movement Patterns That Can be Performed

In the previous section, we pointed out that there is a limit to the notion of assuming a single movement pattern that is optimal in terms of achieving the best movement outcome as the reference movement pattern for assessing the fitness for purpose of a movement technique. That is, it is better to consider that there may be more than one movement pattern that would actually be performed when a certain movement technique is employed. Among the possible movement patterns, there must be some that occur with high frequency and some that occur only rarely (or not at all). In such a scenario, it seems to be a generally applicable notion to use weighted averaging of the movement outcomes of the multiple movement patterns that can be performed according to the probability of the occurrence of each pattern. In this manner, the approach discussed in the previous section can also be considered as a special case where a probability of 1 is assigned to the optimal movement pattern.

Consider a hypothetical simulation study using a musculoskeletal model. As this is merely an example to make our discussion easier to understand, we shall assume that fluctuations in movement patterns when the same motor task is repeated arises from noise added to a single neural input pattern. We can obtain a typical movement pattern for a certain motor task from a skilled player and identify the neural input pattern that replicates the measured movement pattern as much as possible using, for example, a tracking method (Lin and Pandy, 2017). Assuming the effects of noise in the neural stimulation (Faisal et al., 2008), by adding perturbations based on given probability distributions to the neural inputs of each muscle from time to time, we can simulate movement patterns that occur when the model is given a neural input pattern that deviates slightly from the first one estimated. By repeating this procedure for a sufficient number of cycles, we can obtain the probability distribution of multiple movement patterns and their associated movement outcomes. By conducting this procedure when two different movement techniques are employed, it may be possible to assess the fitness for purpose of a certain movement technique.

For the convenience of embodying our thoughts, we have provided the above example using a hypothetical simulation study. However, considering the technical limitations of the current simulation study, as mentioned in the previous section, a practical investigation using experimental and observational methods would be necessary in most cases. In this regard, it appears that it would be sufficient to collect data for each group that habitually employs different movement techniques and simply compare the performance among those groups (Elliott et al., 1997). This is because it seems convincing to argue that the set of movement patterns performed by actual players is nothing but the set of movement patterns that could be performed if a certain movement technique is employed.

To address this issue, a more refined definition of executable movement patterns should be considered. One possible interpretation is that the set of movement patterns that can be achieved when an athlete reaches his/her full potential are the “movement patterns that can actually be performed.” This definition leads to the notion of assessing the properties of a certain movement technique based on the ultimate performance that humans can achieve when employing said technique. Ultimately, perhaps we should assess the fitness for purpose of movement techniques based on this criterion. However, the set of movement patterns that follow this criterion is not something that researchers can observe. This is because when considering the movement patterns that can be performed when humans reach their full potential, it is necessary to assume, for example, that they have mastered a knack for using a certain movement technique that is currently unknown to anyone else.

Another possible interpretation is to consider the set of movement patterns that can be performed when the overall movement patterns are adjusted to a movement technique over a sufficiently long period of time in the present as “movement patterns that can actually be performed.” We believe that the approach of comparing the movement patterns and outcomes of two separate populations that habitually employ two different movement techniques can be expected to be reasonably useful in assessing the fitness for purpose of the movement techniques according to this second criterion.

From the standpoint of observability, motion analysis researchers will, to some extent, be forced to conduct their studies according to the second criterion to grasp the fitness for purpose of movement techniques. An important point that can be clarified *via* contrast with the first criterion is that the fitness for purpose of movement techniques assessed based on the second criterion is not immutable, but may vary with time. This is because it is assumed that practical knowledge in performing a certain motor task will be improved daily through the efforts of many athletes, coaches, and researchers.

## Discussions

In this paper, we have developed a conceptual analysis of the type of framework that should be used to assess the fitness for purpose of movement techniques in motion analysis research. However, researchers, especially those who hold the dynamical systems approach opinion, may have the impression that it is no longer natural to have a purpose or constraint perspective. They may also feel that such a framework has already been established. Of course, we fully appreciate the value of the framework proposed, for example, by Newell (1986) and Newell and Jordan (2007). However, as motor tasks become more and more complex, identifying the goals and constraints, knowing what is the optimal behavior for the goals and constraints, or discovering and executing the optimal behavior for the goals and constraints by the actors become difficult. Analysis at the biomechanics level may help find the goals and constraints of behavior in such situations. However, it should be noted that it is not easy to grasp what the behavior is for using biomechanical data. Bernstein previously pointed out some cases in which a course of action contrary to the end result, such as the need to tighten a belt before loosening it, is fit for achieving the desired end result (Bernstein, 1996). As mentioned in section Requirements Specific to Motion Analysis Research When Considering Fitness for Purpose, biomechanical motion analysis is not very good at assessing fitness for purpose in such cases. In view of this point, what we have presented is a framework in which it is possible to extract the “purpose” of an action in situations where it is not clear what the end goal of the action is.

The framework we have presented is characterized by its duality of objectivism and relativism. Interpreting the question “What is the purpose of a certain movement technique?” based on, for example, subjective intentions of athletes, makes it difficult for biomechanical motion analysis researchers to relate it logically to commonly available data. The framework we have proposed attempts to slightly reinterpret the meaning of such purposive questions, thereby opening up the possibility of a logical discussion within the information of the mechanical dimension. In this sense, an aspect of our framework is oriented toward a kind of objectivism. Note that this assertion is not to say that only the biomechanical dimension is useful in discussing the purpose of movement techniques. For example, research that investigates the intentions of the actors rather than the movements at the level of individual muscles and joints, which is what we mainly assume, remains a powerful means of approaching the purpose of movement techniques. We would rather expect to strengthen the complementarity with such a hierarchy of actions. For example, when an actor describes what his/her movement technique is for, our framework will contribute by providing criteria for what data should accompany the biomechanical dimension to assert that it is not based on a misunderstanding.

According to our discussion, the fitness for purpose of a movement technique is a concept wherein the assessment varies if the movement technique being compared or the context being assumed differ. Whether or not a certain movement technique is fit for purpose should be considered relative to what is assumed as desired results, the physical characteristics of the person employing it, and the environment in which it is used. In this regard, our framework is based on relativism or contextualism. This perspective clarifies that the notion that statistical evidence for reproducible phenomena among multiple subjects is a prerequisite for scientific research does not work well for the analysis of movement techniques in some situations. For example, it helps us to smoothly understand that a movement technique that is good on average in a group will not be good if it is based on the physical characteristics of a particular individual. Similarly, it also suggests that when there is a discrepancy between a view based on the overall judgment of a highly trained coach and a view derived from scientific experiments, the overall judgment of a highly trained coach sometimes prevail. The problem of concern is that a movement technique that is fit for purpose under the conditions of special instruction by a superior coach may send the wrong message to that superior coach that “the movement technique you think is good is not that good” if the scientific experiment examines a set that does not meet those limiting conditions, in this case including instruction by mediocre coaches. Recognizing that there is also contextual relativity in the set of movement patterns that arise when a certain movement technique is employed will allow us to be moderately skeptical of the results of scientific experiments in such cases. We argue that motion analysis researchers need to be aware that the topics they are interested in inherently contain such contextual relativity.

Our framework can be regarded as a refinement of the objective criteria for determining whether a certain movement technique is fit for purpose. The discussion thus far has mainly referred only to what difference employing a certain movement technique makes to the eventual movement outcome. However, it may not be sufficient to explore our original questions. What we have developed thus far is merely a framework that enables us to assess whether the employment of a certain movement technique will lead to better movement outcomes, but it does not explain in any convincing way *why* employing this technique will improve the outcome of the motor task. Our future interest is to consider whether extending the notion of analyzing probabilistic relationships between different events, as presented in this paper, can answer the question of why the employment of a certain movement technique increases the probability of a more desirable movement outcome. For example, if it is possible to state, “employing movement technique A increases the probability of the occurrence of event B, which increases the probability of success in the motor task,” then this would explain why employing movement technique A is likely to lead to better outcomes. We believe that the possibility of creating such an explanation needs to be subjected to the same type of conceptual analysis outlined in this paper.

In conclusion, we would like to emphasize that there are many important issues for motion analysis researchers in topics such as those dealt with in this paper. Our discussion suggests that answering the question of what a certain movement technique is for, that is, what is the fitness for purpose of a certain movement technique, may be a unique challenge that is peculiar to motion analysis research. If this is the case, we motion analysis researchers need to establish our own reasoning scheme that is well-suited to the nature of our problem. We believe that we have presented an argument that can serve as a starting point for developing a theoretical framework for interpreting the meaning of behavior in motion analysis research. We hope that our discussion has shown the necessity and utility of a certain type of philosophical inquiry in developing this research domain.

## Author Contributions

LO, SF, and SY conceived, designed the work, and revised the manuscript. LO drafted the manuscript. All authors contributed to the article and approved the submitted version.

## Funding

This work was supported by a Grant-in-Aids for JSPS (Tokyo, Japan) Research Fellow grant number 18J22999.

## Conflict of Interest

The authors declare that the research was conducted in the absence of any commercial or financial relationships that could be construed as a potential conflict of interest.

## Publisher's Note

All claims expressed in this article are solely those of the authors and do not necessarily represent those of their affiliated organizations, or those of the publisher, the editors and the reviewers. Any product that may be evaluated in this article, or claim that may be made by its manufacturer, is not guaranteed or endorsed by the publisher.
